# Potential importance of protease activated receptor (PAR)-1 expression in the tumor stroma of non-small-cell lung cancer

**DOI:** 10.1186/s12885-017-3081-3

**Published:** 2017-02-07

**Authors:** Cong Lin, Christof J. Majoor, Joris J. T. H. Roelofs, Martijn D. de Kruif, Hugo M. Horlings, Keren Borensztajn, C. Arnold Spek

**Affiliations:** 1 Center for Experimental and Molecular Medicine, Academic Medical Center, Amsterdam, 1105 AZ The Netherlands; 2 Department of Respiratory Medicine, Academic Medical Center, Amsterdam, 1105 AZ The Netherlands; 3 Department of Pathology, Academic Medical Center, Amsterdam, 1105 AZ The Netherlands; 4 Department of Pulmonology, Zuyderland Hospital, Henri Dunantstraat 5, 6419 PC Heerlen, The Netherlands; 5 Department of Pathology, The Antonie van Leeuwenhoek hospital, Amsterdam, 1066 CX The Netherlands; 6 Inserm UMR1152, Medical School Xavier Bichat, 16 rue Henri Huchard, 75018 Paris, France; 7 Département Hospitalo-universtaire FIRE (Fibrosis, Inflammation and Remodeling) and LabEx Inflamex, Paris, France

**Keywords:** Protease activated receptor, NSCLC and tumor stroma

## Abstract

**Background:**

Protease activated receptor (PAR)-1 expression is increased in a variety of tumor cells. In preclinical models, tumor cell PAR-1 appeared to be involved in the regulation of lung tumor growth and metastasis; however the role of PAR-1 in the lung tumor microenvironment, which is emerging as a key compartment in driving cancer progression, remained to be explored.

**Methods:**

In the present study, PAR-1 gene expression was determined in lung tissue from patients with non-small-cell lung cancer (NSCLC) using a combination of publicly available RNA microarray datasets and in house-made tissue microarrays including tumor biopsies of 94 patients with NSCLC (40 cases of adenocarcinoma, 42 cases of squamous cell carcinoma and 12 cases of other type of NSCLC at different stages).

**Results:**

PAR-1 gene expression strongly correlated with tumor stromal markers (i.e. macrophage, endothelial cells and (myo) fibroblast markers) but not with epithelial cell markers. Immunohistochemical analysis confirmed the presence of PAR-1 in the tumor stroma and showed that PAR-1 expression was significantly upregulated in malignant tissue compared with normal lung tissue. The overexpression of PAR-1 in tumor stroma of NSCLC appeared to be independent from tumor type, tumor stage, histopathological differentiation status, disease progression and patient survival.

**Conclusion:**

Overall, our data provide evidence that PAR-1 in NSCLC is mainly expressed on cells that constitute the pulmonary tumor microenvironment, including vascular endothelial cells, macrophages and stromal fibroblasts.

**Electronic supplementary material:**

The online version of this article (doi:10.1186/s12885-017-3081-3) contains supplementary material, which is available to authorized users.

## Background

Lung cancer is the leading cause of cancer related death, with around 1.6 million deaths worldwide and the mortality rates for lung cancer are still increasing annually [1, 2]. Non-small-cell lung cancer (NSCLC), the most common type of lung cancer, has a devastating survival outcome. Traditional chemotherapy, including predominantly platinum-based regimens, as first-line standard treatment for NSCLC only shows a modest prolongation of median and overall survival. Despite aggressive multimodality therapy, 5-year survival rate for patients with stage IV NSCLC at diagnosis is only approximately 2% [3]. More recently, targeted therapies showed efficacy in patients with advanced NSCLC who have specific genetic alterations, like mutations of the anaplastic lymphoma kinase gene or of the epidermal growth factor receptor [1]. However, these available molecular therapies can only be applied to selective patients and the observed benefits are small, suggesting that more in-depth studies of molecules that relate to the pathogenesis of NSCLC is required.

Protease-activated receptor (PAR)-1 is a cell surface seven-transmembrane G protein coupled receptor that is activated by proteolytic cleavage. Removal of the N-terminal extracellular domain of PAR-1 reveals a new tethered ligand that binds to the body of PAR-1 and activates transmembrane signaling to intracellular G proteins, thereby leading to multiple pathophysiological responses [4, 5]. Overexpression of PAR-1 has been detected in various types of cancers, including ovarian, breast, lung, prostate cancer and melanoma [6–10]. Importantly, elevated PAR-1 expression is closely associated with diseases progression and overall survival in breast, prostate, gastric cancer and melanoma [6, 8, 9, 11]. Moreover, tumor cell PAR-1 is recently identified as a promising target to decrease lung cancer progression. Indeed, PAR-1 pepducin inhibitors not only block the migration of both primary and established lung cancer cell lines, but also significantly limit lung tumor growth in nude mice [10]. Moreover, melanoma growth and metastasis were significantly decreased in mice treated with PAR-1 small interfering RNA (siRNA) [12].

During the last decade, the paradigm that tumor growth solely relies on the malignant cells has shifted to a more comprehensive view that tumor growth is dependent on interactions between cancer cells and their adjacent microenvironment, also known as the stroma [13]. The tumor stroma, predominately composed of basement membrane, fibroblasts, vasculature with endothelial cells, inflammatory cells and extra cellular matrix proteins such as collagen and fibronectin [14], is indeed emerging as a key player in promoting carcinogenesis by modulating tumor growth, angiogenesis, invasion and metastasis [15, 16]. Targeting the tumor stroma is consequently under intense investigation as novel treatment strategy in cancer.

Interestingly, PAR-1 expression is not tumor cell specific and PAR-1 is also expressed on key cell types that constitute the tumor stroma such as endothelial cells, fibroblasts and macrophages. Activation of PAR-1 on these stromal cells leads to increased vascular permeability, fibroblast activation, extracellular matrix production and cytokine secretion, thereby potentially driving tumor growth and metastasis [13]. In line with these observations, colonic adenocarcinoma growth was limited in PAR-1-deficient mice, suggesting the importance of PAR-1 in the tumor microenvironment [17]. In addition, pancreatic tumors in PAR-1 deficient animals were significantly smaller compared with tumors in wild type mice. Moreover, the same study also showed that stromal cells drive tumor growth and induce chemoresistance of pancreatic cancer in a PAR-1 dependent manner [18]. Overall these data point to an important role of stromal cell-associated PAR-1 in tumor progression. However, the role of stromal PAR-1 in lung cancer has not been explored yet. In the present study, we examined PAR-1 expression in NSCLC stroma and assessed its correlation with disease progression.

## Methods

### Patients

Tissue microarrays (TMAs, triplicate cores per case) were prepared with tumor sections obtained from NSCLC patients during surgery according to the guidelines of the Medical Ethical Committee of the Academic Medical Center of Amsterdam. The TMAs consist of samples from 94 patients with NSCLC, including 40 cases of adenocarcinoma (ADC), 42 cases of squamous cell carcinoma (SCC) and 12 cases of other type of NSCLC at different stages (Table 1). On each TMA, 3 cases of healthy lung tissue (i.e. adjacent normal tissue) were also included.

**Table 1:** Patient characteristics

Characteristic	Patient	
	N	%
Male	63	67
Median Age (Range)	66	
	(30–86)	
Progression	26	36
Tumor type:		
Adenocarcinoma	40	42.5
Squamous cell carcinoma	42	44.7
Other type*	12	12.8
Tumor differentiation:		
Well differentiated	6	10.4
Less differentiated	30	51.7
Little differentiated	14	24.1
Poorly differentiated	8	13.8
NSCLC stage:		
I	53	57.6
II	28	30.4
III	10	10.9
IV	1	1.1
Lymph node metastasis	14	15.2

*This group includes 2 large cell carcinoma patients, 10 patients with mixed tumor types (for instance adenocarcinoma/bronchioloalveolar carcinoma)

### Mining of publically available RNA microarray dataset

The datasets were derived from Gene Expression Omnibus (http://www.ncbi.nlm.nih.gov/gds) using the R2 microarray analysis and visualization platform (http://r2.amc.nl). Correlation of gene expression between PAR-1 and markers of different stromal cell types in NSCLC cancer patients were derived by the R2 program from five different datasets, including Bild (*n* = 114, GSE3141), Peitsch (*n* = 150, GSE43580), EXPO (*n* = 121, GSE2109), Mao (*n* = 124, GSE 31852) and Hou (*n* = 156, GSE 19188).

### Immunohistological analysis

Four-μm sections were first deparaffinized and rehydrated. Endogenous peroxidase activity was quenched with 0.3% H2O2 in methanol. PAR-1 staining was performed with a primary antibody specific for PAR-1 (ATAP-2 ;1:200; SC-13503, 24 h at 4 °C, Santa Cruz, San Diego, CA) [19, 20]. A horseradish peroxidase-conjugated polymer detection system (ImmunoLogic, Duiven, the Netherlands) was applied for visualization, using an appropriate secondary antibody and diaminobenzidine staining. Specimens with PAR-1 immunostaining were reviewed jointly at a multi-head microscope by 2 investigators blinded to the patients’ clinical status. To evaluate immunohistochemical expression of PAR-1, the intensity of PAR-1 staining was graded by consensus on a scale from 0 to 3 (0 = negative staining; 1 = weakly positive; 2 = moderately positive; 3 = strongly positive). Slides were photographed with a microscope equipped with a digital camera (Leica CTR500).

### Statistics

Statistical analyses were conducted using GraphPad Prism (GraphPad software, San Diego). Comparisons between conditions were analyzed using two tailed unpaired t-tests when the data were normally distributed; otherwise Mann–Whitney analysis was performed. Results are expressed as mean ± SEM, *P* values < 0.05 are considered significant.

## Results

### PAR-1 gene expression is correlated with lung tumor stroma activation

**Fig. 1:**
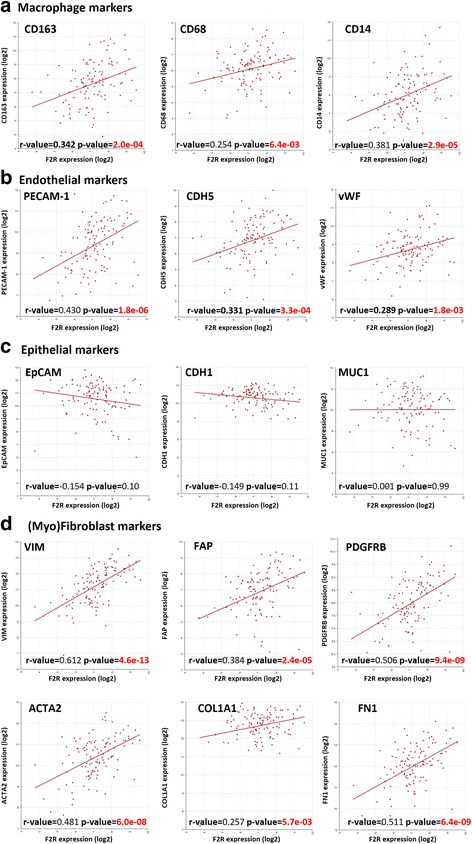
PAR-1 expression correlates with stromal markers in NSCLC patients. Scatter plot of PAR-1 gene (F2R) expression versus the expression of specific macrophage (**a**), endothelial (**b**), epithelial (**c**) and (myo) fibroblast (**d**) markers in tumors derived from NSCLC patients (Bild microarray dataset; GSE3141, *n* = 114). Linear regression analysis was used to determine the correlation coefficient, and *p*-values of significant correlations are indicated in *red*

To explore the association of PAR-1 expression with the NSCLC stroma, we correlated PAR-1 gene expression levels with specific markers of different stromal cell types, including macrophages, endothelial cells, epithelial cells and (myo) fibroblasts in resected tumor specimens using publicly available microarray datasets. To this end, 3 markers were selected for each stromal cell type, except for (myo) fibroblasts for which we included markers of differentiated fibroblasts and markers for extracellular matrix (ECM) produced by myofibroblasts. Interestingly, tumors with higher PAR-1 levels also displayed elevated expression levels of markers for macrophages, endothelial cells and (myo) fibroblasts on the microarrays. Using the GSE3141 dataset (Fig. 1), PAR-1 gene expression was correlated with all three markers for human monocytes and macrophages, i.e. *CD68* (*p* < 0.01)*, CD163* (*p* < 0.001) and *CD14* (*p* < 0.0001) [21]. Correlations with specific vascular endothelial cell markers (e.g. Platelet endothelial cell adhesion molecule (*PECAM)-1*) and fibroblasts markers (e.g. Vimentin (*VIM)* and fibroblast activation protein alpha *(FAP*)) were also significant (*p* < 0.0001), with *r*-values ranging from 0.2 to 0.7. The commonly used differentiation marker for fibroblasts *ACTA2* (gene encoding for alpha-smooth muscle actin, α-SMA [22]) and markers for prominent constituents of ECM deposition Collagen, type I, alpha (*COL1A1)* and Fibronectin *(FN1)* were also all correlated with PAR-1 gene expression in the NSCLC specimens (all *p* < 0.01). Intriguingly, PAR-1 expression did not correlate to epithelial (tumor) cell markers Epithelial cell adhesion molecule (*EpCAM)*, Cadherin 1 (*CDH1)* and Mucin 1 (*MUC1)*. These observed correlations (and lack of correlation in epithelial cells) were confirmed in four additional independent microarray datasets from NSCLC (Table 2). However, no correlation between PAR-1 and stromal markers was observed in the healthy control group included in the Hou *et al.* set (GSE19188), suggesting the correlation between PAR-1 gene expression and stroma activity specifically exists in tumor microenvironment. To confirm the identity of the stromal cell types expressing PAR-1, we performed immunohistochemistry with different cell type markers on consecutive lung cancer slides. As shown in Additional file 1: Figure S1, PAR-1 positive areas are also positive for CD31 (endothelial marker), CD68 (macrophage marker) and aSMA (myofibroblast marker).

**Table 2:**
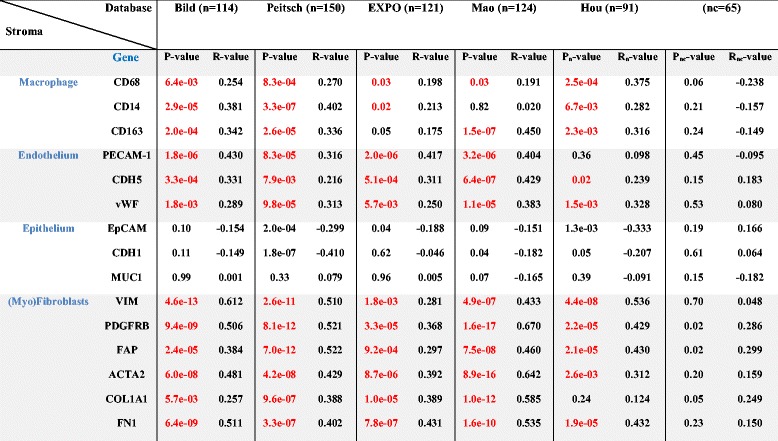
Correlation of gene expression between PAR-1 and markers of different stromal cell types

Selection of the datasets is based on the patient group size ≥50. Bild (*n* = 114, GSE3141), Peitsch (*n* = 150, GSE43580), EXPO (*n* = 121, GSE2109), Mao (*n* = 124, GSE 31852), Hou (*n* = 91, control group nc = 65, GSE 19188). Linear regression analysis was used to determine *p*-value of correlation. Significant correlations are indicated in red

### PAR-1 is overexpressed in stroma of primary pulmonary tumors on TMAs

**Fig. 2:**
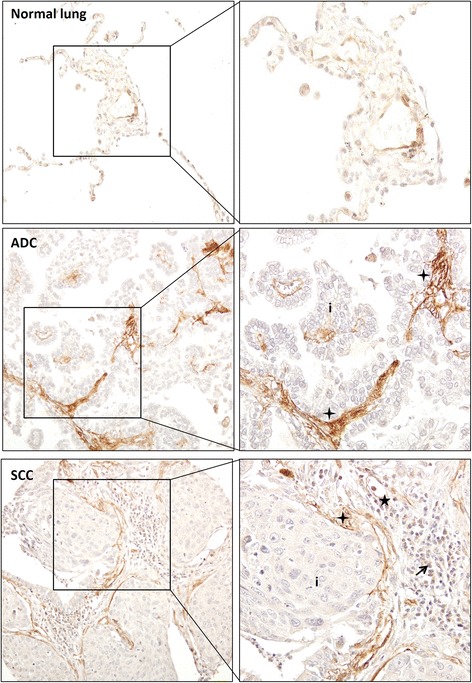
Stromal PAR-1 expression is upregulated in NSCLC patients. Representative PAR-1 staining of normal lung tissue and tumor sections of NSCLC patients (Pictures were taken with 100x magnification; Enlarged pictures were taken with 200x magnification). ADC indicates adenocarcinoma, SCC indicates squamous cell carcinoma and LCC indicates large cell carcinoma. Tumor cells are indicated by (i) whereas inflammatory cells are indicated by *solid arrowheads*, vascular endothelial cells are indicated by stars and fibroblasts-like cells and ECM are indicated by *crosses*

**Fig. 3:**
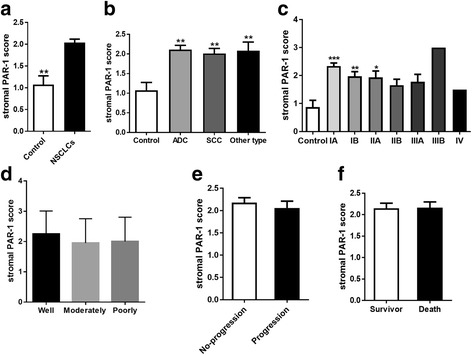
Association of stromal PAR-1 expression with clinical parameters in NSCLC patients. **a** Stromal PAR-1 expression in healthy lung tissue and in NSCLC. **b** Stromal PAR-1 expression in healthy lung tissue and in different types of NSCLC. **c** Stromal PAR-1 expression in healthy lung tissue and in different stages of NSCLC. **d** Stromal PAR-1 expression according to the differentiation status of NSCLC, including well differentiated, moderately differentiated and poorly differentiated. **e** Stromal PAR-1 expression in NSCLC patients with disease progression and in patients with stable disease (no-progression). **f** Stromal PAR-1 expression of survivors and non-survivors of NSCLC. All data are expressed as mean ± SEM, *P < 0.05, **P < 0.01 and *** *P* < 0.001

To confirm the presence of PAR-1 in NSCLC stroma, we next analyzed PAR-1 protein expression in tumor sections using immunohistochemistry. Ninety-four patients with pathologically confirmed diagnosis of NSCLC were included into this study. The median age at diagnosis was 66 years (range 30 to 86 years), and the majority of patients had NSCLC stage I disease (*n* = 53, 57.6%). Six cases were well differentiated (2 ADC, 1 SCC, 3 other types), 30 cases were moderately differentiated (12 ADC, 18 SCC) and 22 cases were poorly differentiated (10 ADC, 11 SCC, 1 other types) (Table 1). Overall, strong PAR-1 expression was seen in stroma of all different types of NSCLC (ADC, SCC and large-cell carcinoma) as opposed to weak PAR-1 staining on control sections (Fig. 2). In line with our observations in the tumor microarray datasets, the stromal cells (fibroblast-like cells, inflammatory cells and endothelial cells) were all intensively stained for PAR-1, while cancer cells were negative for PAR-1 or showed only weak PAR-1 staining. Subsequent quantifications showed that 93 out of the 94 cases had PAR-1 expression in the stroma, with an average score of 2, while 1 SSC patient was PAR-1 negative. Importantly, the average PAR-1 score in control lungs was significantly lower as in NSCLC stroma (average score of 1; Fig. 3a). As shown in Fig. 3b, PAR-1 levels were similar in different subtype of NSCLC (average scores of 2.11, 2.01 and 2.08 for ADC, SCC and other type of NSCLCs respectively). Stromal PAR-1 expression levels did not correlate with clinical variables like stage of NSCLC (Fig. 3c), differentiation status (Fig. 3d), disease progression (Fig. 3e) and overall survival (Fig. 3f).

## Discussion

One of the anticipated future treatment options for NSCLC is to target the interactions between tumor and stromal cells, since stromal cells provide additional signals that support tumor growth and invasion [1, 16]. In the present study, we determined PAR-1 expression in NSCLC patients and found high PAR-1 expression predominantly in the tumor stroma compartment during early stage cancer. This was reflected by the correlation of PAR-1 gene expression with stroma markers like CD163, CD31 and vimentin, and ECM proteins like collagen and fibronectin, as well as by a significant increase in the intensity of PAR-1 staining in stromal cells of tumor tissue compared with normal lung tissue. Although it has been documented that upregulation of PAR-1 expression appears in a variety of invasive cancers of epithelial origin, our data do show that increased PAR-1 expression in NSCLC patients arises mainly in the tumor stroma rather than in the epithelial cancer cells.

The observed PAR-1 expression pattern in NSCLC resembles that seen in other malignancies. In breast cancer, PAR-1 expression, as shown by immunohistochemistry and *in situ* hybridization, is observed in mast cells, macrophages, endothelial cells, and vascular smooth muscle cells of the metastatic tumor microenvironment. Interestingly however, PAR-1 expression is particularly increased in stromal fibroblasts surrounding breast carcinoma cells as opposed to low/negative expression in fibroblasts of healthy or benign conditions [23]. Moreover, in prostate cancer PAR-1 is predominantly expressed in peritumoral stroma. In particular, PAR-1 is mainly expressed in myofibroblasts and to a lower level in endothelial cells in isolated capillaries around the malignant glands [24, 25].

The enrichment of PAR-1 expression in the stroma surrounding the tumor may actually be clinically relevant. Indeed, in the setting of pancreatic cancer, PAR-1 also coincides with the expression pattern of the stromal markers, such as vimentin, collagen I and α-SMA [18]. More importantly, PAR-1 promoted monocyte recruitment due to fibroblast dependent chemokine production, thereby driving pancreatic tumor growth and chemoresistance [18]. In the context of lung cancers, the expression of PAR-1 mRNA in alveolar walls with surface spreading of neoplastic cells was shown to increase by 10-fold compared with alveolar walls without surface spreading of neoplastic cells, and stimulation of PAR-1 led to the proliferation of alveolar capillary endothelial cells, pointing to PAR-1 as a potential regulator in alveolar angiogenesis [26]. Interestingly, accumulating evidence indicates that PAR-1 also exerts pro-inflammatory and pro-fibrotic functions through macrophages and fibroblasts during pulmonary fibroproliferative disease progression [27–29], which may also benefit tumor progression and metastasis.

Previous studies about PAR-1 in NSCLC focused on its function in cancer cells. Indeed, multiple reports showed that PAR-1 modulates lung cancer cell proliferation and migration, thereby supporting tumor growth and invasion [10, 30]. Hence, targeting PAR-1 to inhibit progression of lung cancer cells seems to be an option for cancer therapy. Recently, emphasis has shifted toward the tumor stroma for novel therapeutic strategies and several approaches targeting the stromal tissue in different types of cancers have been proved to be effective [31–33]. Our data showing high stromal PAR-1 expression in NSCLC may thus indicate stromal PAR-1 may be the main target of the treatment for NSCLC. However, before drawing conclusions on potential clinical implications of stromal PAR-1 in NSCLC, it is important to elucidate the functional consequence of PAR-1 activation on stromal cells with respect to lung cancer development.

In the present study, we observed that PAR-1 expression is highly upregulated in the tumor stroma but not in normal lung tissue, suggesting that PAR-1 may have a diagnostic value in NSCLC. However, the increased PAR-1 expression does not seem to correlate with diseases progression, which indicates that stromal PAR-1 in lung cancer is crucial for carcinogenesis but may not be a determinant factor for cancer progression. These results are in line with a recent study by Erturk and colleagues, who determined serum PAR-1 levels in 80 patients with lung cancer [34]. Serum PAR-1 concentrations of lung cancer patients were significantly increased as compared to controls (i.e. median values of 26.45 ng/mL and 0.07 ng/mL, respectively), but serum PAR-1 levels did not correlate with clinical variables and failed to predict prognosis of the lung cancer patients. In apparent disagreement, other studies using immunohistochemistry analysis showed that PAR-1 may be a prognostic factor for poor prognosis in both early-stage and advanced stages (III and IV) of NSCLC [35, 36]. Importantly however, these studies analyzed tumor cell PAR-1 expression and did not address PAR-1 expression in the stromal compartment.

## Conclusion

In summary, our data show PAR-1 is overexpressed in the tumor stroma of NSCLC, but stromal PAR-1 expression levels do not correlate with disease progression and/or overall survival.

## Additional file

Additional file 1: Figure S1. Correlation of PAR-1 expression and specific markers for endothelial cells, macrophages and myofibroblasts. Consecutive lung cancer slides stained for PAR-1 (left panels), CD31 (endothelial marker), CD68 (macrophage marker) and aSMA (myofibroblast marker). Please note that due to the use of consecutive slides, the structure of the tissue in the PAR-1 stained slide is somewhat different from the CD31, CD68 and aSMA stained slides. Pictures were taken with 100x magnification. (TIF 7026 kb)

## Abbreviations

ADC: Adenocarcinoma
CDH1: Cadherin-1
COL1A1: Collagen, type I, alpha
ECM: Extracellular matrix
EpCAM: Epithelial cell adhesion molecule
FAP: Fibroblast activation protein alpha
FN1: Fibronectin 1
MUC1: Mucin 1
NSCLC: Non-small-cell lung cancer
PAR-1: Protease activated receptor-1
PECAM-1: Platelet endothelial cell adhesion molecule-1
SCC: Squamous cell carcinoma
siRNA: Small interfering RNA
TMA: Tissue microarray
VIM: Vimentin
α-SMA: Alpha-smooth muscle actin
