# Supplementary figures and images for: Potential importance of protease activated receptor (PAR)-1 expression in the tumor stroma of non-small-cell lung cancer
Source: BMC Cancer. 2017 Feb 7;17:113. doi: 10.1186/s12885-017-3081-3 (PMC5297223; doi:10.1186/s12885-017-3081-3)

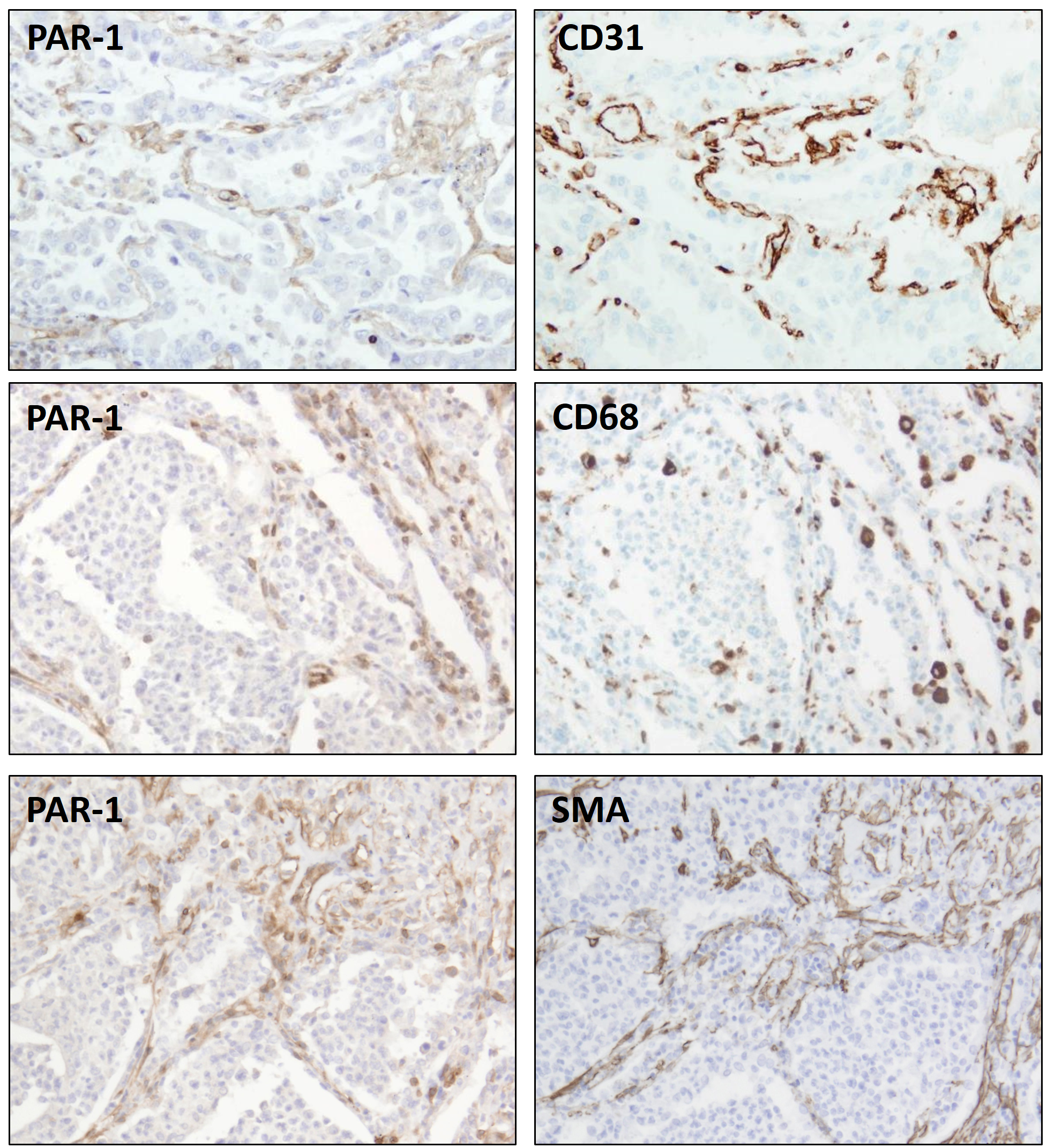

Supplement: Additional file 1: Figure S1. — Correlation of PAR-1 expression and specific markers for endothelial cells, macrophages and myofibroblasts. Consecutive lung cancer slides stained for PAR-1 (left panels), CD31 (endothelial marker), CD68 (macrophage marker) and aSMA (myofibroblast marker). Please note that due to the use of consecutive slides, the structure of the tissue in the PAR-1 stained slide is somewhat different from the CD31, CD68 and aSMA stained slides. Pictures were taken with 100x magnification. (TIF 7026 kb) [file 12885_2017_3081_MOESM1_ESM.tif]
